# Management of oral *Pseudomonas aeruginosa* infections in oncology patients using antimicrobial photodynamic therapy, photobiomodulation, and systemic antibiotics: a case series

**DOI:** 10.31744/einstein_journal/2026AO2422

**Published:** 2026-07-29

**Authors:** Marcella Ferreira Gobbi, Fernanda de Paula Eduardo, Nidia Castro dos Santos, Giovana Ferrari Molina, Patrick Polcaro de Paiva Santos, Danielle Lima Corrêa de Carvalho, Leticia Mello Bezinelli

**Affiliations:** 1 Universidade de São Paulo Faculdade de Odontologia São Paulo SP Brazil Faculdade de Odontologia, Universidade de São Paulo, São Paulo, SP, Brazil.; 2 Hospital Israelita Albert Einstein Faculdade Israelita de Ciências da Saúde Albert Einstein São Paulo SP Brazil Faculdade Israelita de Ciências da Saúde Albert Einstein, Hospital Israelita Albert Einstein, São Paulo, SP, Brazil.

**Keywords:** Photochemotherapy, Low-level light therapy, Pseudomonas aeruginosa, Oral infections, Neoplasms, Immunocompromised host, Anti-infective agents

## Abstract

**Objective:**

To describe a case series of oral *Pseudomonas aeruginosa* infections in oncology patients managed using combined antimicrobial photodynamic therapy and photobiomodulation in association with systemic antibiotic therapy guided by microbiological culture and antibiogram.

**Methods:**

Nine oncology patients with culture-confirmed oral *Pseudomonas aeruginosa* infections were treated with alternating sessions of antimicrobial photodynamic therapy and photobiomodulation combined with systemic antibiotic therapy selected according to antibiogram results. Clinical response, healing time, and microbiological findings were evaluated.

**Results:**

All patients achieved complete resolution of oral lesions, with a median healing time of 5 days (range, 3-16 days). Microbiological analysis demonstrated high resistance rates to fluoroquinolones and cephalosporins, whereas all isolates remained sensitive to aminoglycosides. No adverse effects or recurrences were observed during follow-up.

**Conclusion:**

Combined antimicrobial photodynamic therapy and photobiomodulation associated with systemic antibiotic therapy guided by culture and antibiogram proved to be a safe, rapid, and reproducible adjunctive approach for managing multidrug-resistant oral infections in oncology patients. Controlled clinical trials are warranted.

## In Brief

Gobbi et al. report the first case series combining antimicrobial photodynamic therapy (aPDT) and photobiomodulation (PBM) with systemic antibiotics for oral *Pseudomonas aeruginosa* infections in oncology patients. All nine patients achieved complete healing (median, 5 days), supporting this multimodal approach for multidrug-resistant oral infections.

## Highlights

First case series of aPDT+PBM for oral *P. aeruginosa* in oncology patients.

Complete lesion resolution in all cases (median healing time: 5 days).

High resistance to fluoroquinolones; aminoglycosides remained effective.

Combined light-based therapy is safe even in severe immunosuppression.

## Graphical abstract

**Figure:**
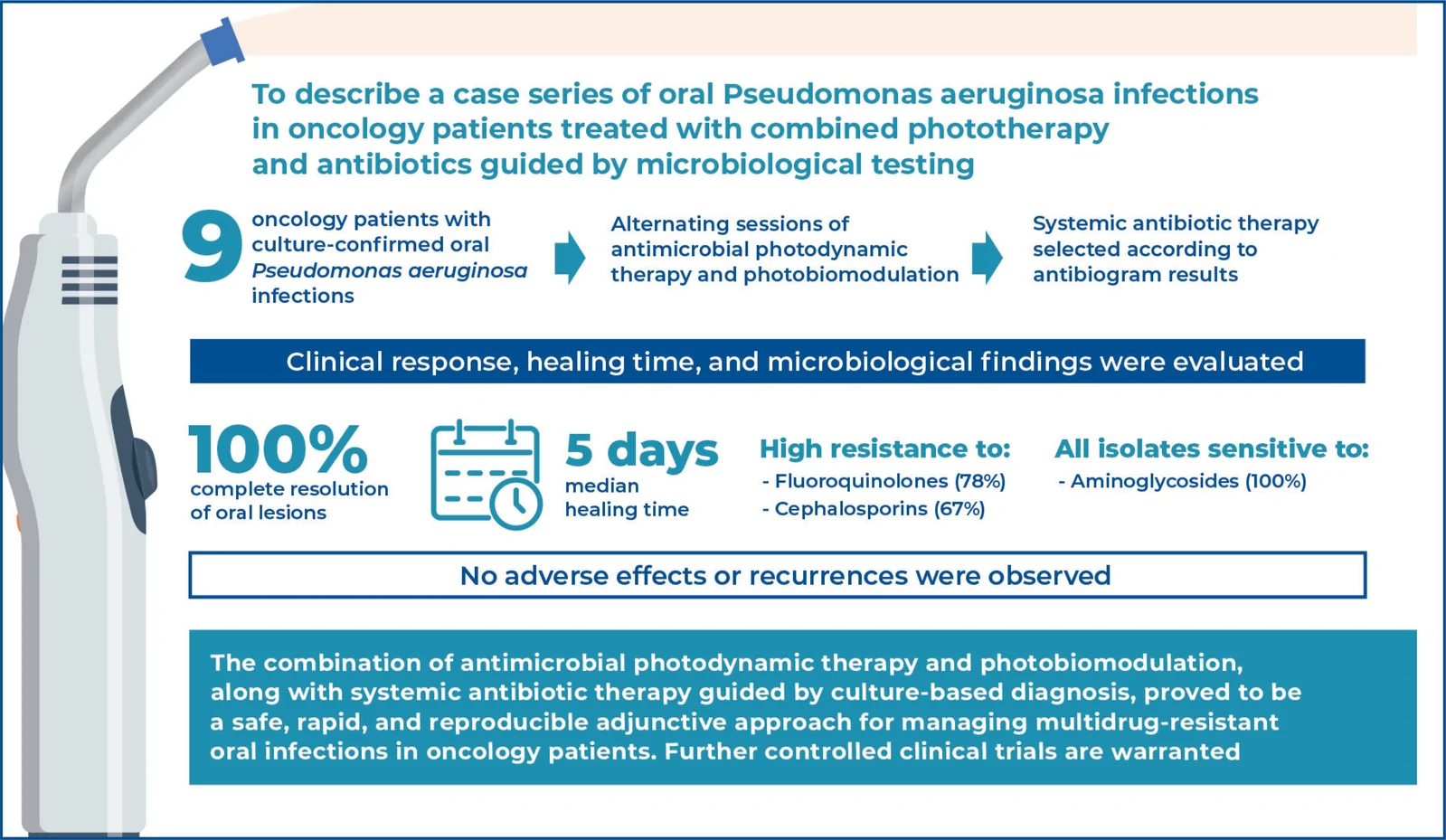

## INTRODUCTION

Opportunistic infections (OIs) caused by *Pseudomonas aeruginosa* are clinically relevant complications in immunocompromised oncology patients. This nosocomial pathogen exhibits high antimicrobial resistance and biofilm formation capacity, which compromise the effectiveness of conventional antibiotic therapy.^(1–3)^

Cancer patients, especially those undergoing chemotherapy or radiotherapy, often develop oral mucosal lesions due to multiple factors, including immunosuppression, treatment-induced oral mucositis, and OIs such as *P. aeruginosa.*^(4)^ These systemic conditions contribute to delayed healing and prolonged symptoms. Patients receiving these treatments are particularly susceptible to OIs because of mucosal damage and immunosuppression.^(4)^

In this context, adjuvant light-based therapies have emerged as valuable tools for controlling OIs and promoting tissue repair. Antimicrobial photodynamic therapy (aPDT) uses a photosensitizer activated by light to reduce microbial load, whereas photobiomodulation (PBM) stimulates tissue repair, with both therapies potentially acting synergistically in oral infection control.^(5,6)^ Although the individual efficacy of aPDT and PBM has been demonstrated in several oral conditions, clinical reports describing their combined use for *P. aeruginosa* infections remain scarce.^(5,7–10)^ The integration of both modalities may enhance microbial control and accelerate mucosal healing when combined with targeted systemic antibiotics.^(5,6)^

Most published reports describe *P. aeruginosa* infections of the skin rather than the oral cavity. Eduardo et al.^(11)^ reported a case of severe oral *P. aeruginosa* infection refractory to systemic antibiotic therapy, in which complete remission occurred only after 3 weeks of consecutive aPDT sessions. Aspiroz et al.^(12)^ described the efficacy of photodynamic therapy with methylene blue for *P. aeruginosa* skin ulcers. Lago et al.^(5,9)^ demonstrated favorable outcomes with combined aPDT and PBM for herpes simplex lesions, whereas Khalil and Hamadah^(10)^ systematically reviewed the association of both therapies for herpes labialis. Despite growing interest in this combined approach, most available publications focus on oral lesions of viral origin, graft-versus-host disease, or medication-related osteonecrosis of the jaw.^(7,8,10–14)^ Although in vitro studies have demonstrated the efficacy of aPDT against *P. aeruginosa* biofilms, clinical evidence for the combined use of aPDT and PBM in oral *P. aeruginosa* infections remains limited.^(13)^

This case series presents nine oncology patients with culture-confirmed oral lesions caused by *P. aeruginosa*, treated with alternating sessions of aPDT and PBM combined with systemic antibiotics guided by antibiogram results, highlighting clinical outcomes and the potential of this combined approach for managing multidrug-resistant oral infections.

## OBJECTIVE

The objective of this study was to report a case series of oncology patients with oral mucosal lesions caused by *P. aeruginosa* treated with antimicrobial photodynamic therapy and photobiomodulation, emphasizing therapeutic protocols, clinical progression of oral lesions, and treatment outcomes.

## METHODS

### Study design

This retrospective observational case series included oncology patients treated at *Hospital Israelita Albert Einstein* (São Paulo, Brazil) between March 2019 and April 2023. Eligible patients had culture-confirmed oral mucosal infections caused by *P. aeruginosa*. Cases were excluded if outcome data were incomplete, if infections involved other *Pseudomonas* species (e.g., *P. monteilli*), or if no cancer diagnosis was present.

The study was approved by the institutional ethics committee of *Hospital Israelita Albert Einstein* (CAAE: 91393825.3.0000.0071; #7.821.006). Because this retrospective study was based on medical records and anonymized clinical data, the Institutional Review Board granted a waiver of informed consent.

### Data collection

Clinical and laboratory data were retrieved from electronic medical records. Lesion scrapings were collected using a standardized oral swab and analyzed cytologically and microbiologically to confirm *P. aeruginosa*. Antibiotic susceptibility testing included amikacin, meropenem, tobramycin, ceftazidime/avibactam, ceftolozane/tazobactam, gentamicin, piperacillin, ciprofloxacin, imipenem, tazocin, cefepime, ertapenem, aztreonam, ampicillin, amoxicillin/clavulanic acid, norfloxacin, and trimethoprim-sulfamethoxazole. Susceptibility was classified as sensitive, intermediate, or resistant according to standard microbiological criteria. Antimicrobial susceptibility testing was performed manually using disk diffusion and microdilution methods. Standardization and interpretation of susceptibility results followed the Brazilian Ministry of Health Ordinance No. 64 (December 2018), which adopts the BrCAST/EUCAST breakpoints. Blood cultures and systemic markers, including white blood cell count, platelets, lymphocytes, and C-reactive protein (CRP), were also recorded.

### Treatment protocols

All patients received systemic antibiotic therapy guided by antibiogram results and local treatment with alternating sessions of aPDT and PBM. The aPDT protocol adopted by the dental team at HIAE consisted of topical application of 0.01% methylene blue gel (Chimiolux, DMC, São Carlos, Brazil) as a photosensitizer. Initially, the affected areas were carefully dried using sterile gauze. The dye was then applied directly to the lesion and allowed to incubate for 5 minutes to ensure adequate penetration. After removal of excess dye with gauze, irradiation was performed. All laser procedures were performed using a low-power continuous-wave diode laser (Therapy XT, DMC, São Carlos, Brazil) emitting red light at a wavelength of 660nm. The device operated at an output power of 100mW with a spot area of 0.09cm², resulting in a power density (irradiance) of 1.1W/cm². The laser beam was applied perpendicularly to the mucosal surface in contact mode, with approximately 1 cm spacing between irradiation points.

For aPDT, light activation was performed for 40 seconds per point, delivering 4 J per point and an energy density (fluence) of 44.4J/cm².^(11)^

For PBM, the same laser device and wavelength were used in continuous mode with identical output power and spot size. Each point received irradiation for 20 seconds, delivering 2J per point and an energy density of 22.2J/cm².^(15)^

The two modalities were applied on alternating days: aPDT sessions focused on microbial reduction, whereas PBM sessions aimed to stimulate tissue repair and analgesia. The number of application points varied according to lesion size and extent, and treatment was maintained in association with systemic antibiotic therapy guided by culture and antibiogram until complete clinical resolution was achieved.

## RESULTS

Nine oncology patients (seven females and two males; mean age 45.0±26.3 years) presented oral mucosal lesions clinically suggestive of bacterial infection. All cases were confirmed by microbiological culture as *P. aeruginosa*. Lesions were located on the gingiva (n=3), palate (n=3), tongue (n=1), and lip (n=2). Five patients presented multiple oral sites, and one patient had bilateral lesions. Most patients were undergoing chemotherapy or radiotherapy at the time of diagnosis and presented clinical signs of mucositis and local pain. Median healing time was 5 days (range, 3-16). Healing time was defined as the interval between the first treatment session and complete re-epithelialization of the oral mucosa, assessed through clinical examination and photographic comparison of lesion progression during treatment. No standardized clinical scale was used for this evaluation. Baseline demographics, lesion sites, and treatment characteristics are summarized in table 1. Antibiotic treatment consisted mainly of meropenem, followed by ciprofloxacin.

**Table 1:** Clinical and therapeutic characteristics of oncology patients with oral mucosal lesions associated with *Pseudomonas aeruginosa*

Patient	Sex	Age	Diagnosis	Lesion sites	aPDT	PBM	Antibiotic	Healing time
1	F	70	Indolent B-cell non-Hodgkin lymphoma with bone marrow infiltration	Gingival mucosa of central incisors, premolars, and molars (upper left side)	Yes	No	Ceftazidime	16 days
2	M	38	Invasive ductal carcinoma of the breast, luminal B	Upper premolar region	Yes	Yes	Cefepime	3 days
3	M	77	Squamous cell carcinoma of the left orbit	Tongue, lips, buccal mucosa, and palate	Yes	Yes	Ciprofloxacin	10 days
4	F	51	Uterine rhabdomyosarcoma with peritoneal metastasis	Soft palate near upper left molars	Yes	Yes	Meropenem	4 days
5	M	15	Hodgkin lymphoma	Gingival mucosa of teeth 23 and 44	No	Yes	Meropenem (IV)	6 days
6	M	27	Acute lymphoblastic leukemia (B-cell lineage)	Gingival mucosa with ulcerative aspect around teeth	Yes	No	Ciprofloxacin	4 days
7	F	48	Acute myeloid leukemia (monocytic)	Gingival mucosa of canines and upper premolars, bilaterally	No	No	Meropenem	6 days
8	F	4	Acute myeloid leukemia and malignant adrenal gland neoplasm	Right side of the palate	No	No	Amikacin sulfate (IV)	6 days
9	F	75	Multiple myeloma	Lower labial mucosa	Yes	Yes	Meropenem	5 days

F: female; M: male; aPDT: antimicrobial photodynamic therapy; PBM: photobiomodulation.

Laboratory findings revealed systemic alterations consistent with infection and immunosuppression. Mean leukocyte count was 5,611±10,513/mm³, and platelet counts averaged 49,554±34,655/mm³, reflecting chemotherapy-induced cytopenia. C-reactive protein levels were elevated in nearly all cases (mean, 117mg/L), and lymphocyte counts were reduced, confirming the patients’ immunocompromised status.

Microbiological cultures demonstrated a consistent pattern of multidrug resistance among the *P. aeruginosa* isolates. The highest resistance rates were observed for fluoroquinolones, particularly ciprofloxacin, and fourth-generation cephalosporins, such as cefepime and ceftazidime. Resistance to β-lactams, including piperacillin-tazobactam, and carbapenems (imipenem and meropenem) was moderate, whereas all isolates remained fully sensitive to aminoglycosides, namely amikacin, gentamicin, and tobramycin. Intermediate susceptibility was observed in fewer than 20% of isolates, primarily for β-lactam combinations. These findings reflect a pattern of broad resistance, particularly to first-line empirical antibiotics.

Blood cultures were performed in eight patients; only two (25%) were positive for *P. aeruginosa*, indicating that infections were predominantly localized to the oral cavity. Representative clinical images are shown in figures 1 and 2, illustrating the healing progression of gingival necrosis in patients with hematologic malignancies treated with combined aPDT, PBM, and systemic antibiotics.

**Figure 1:**
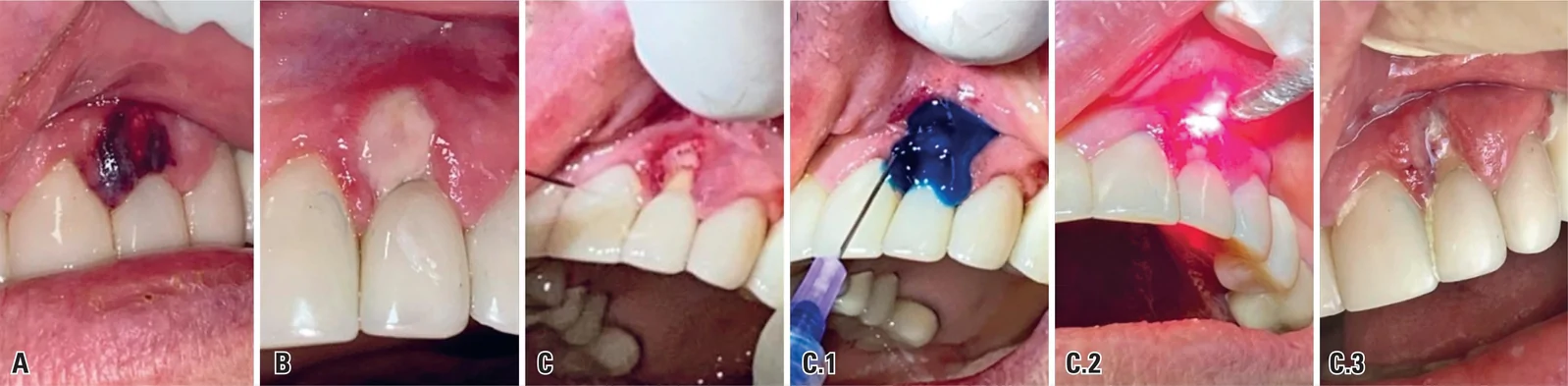
Clinical progression and treatment of a necrotic gingival lesion adjacent to the upper left lateral incisor over a 12-day period in a patient with a history of hematologic malignancy (non-Hodgkin B-cell lymphoma, status post hematopoietic stem cell transplantation). The oral lesion developed approximately 4 months after transplantation during the immunosuppressive recovery phase. (A) Day 0: Initial presentation showing a localized lesion on the attached gingiva with submucosal hematoma. (B) Day 7: Progression to a necrotic appearance with tissue breakdown and fibrinous exudate. (C) Day 11: Surgical debridement of necrotic tissue. (C1) Application of methylene blue photosensitizer (Chimiolux, DMC). (C2) Red light activation (aPDT). (C3) Day 12: Final outcome showing recovered gingival tissue without bacterial colonization, with localized irreversible tissue loss as a sequela of P. *aeruginosa* infection

**Figure 2:**
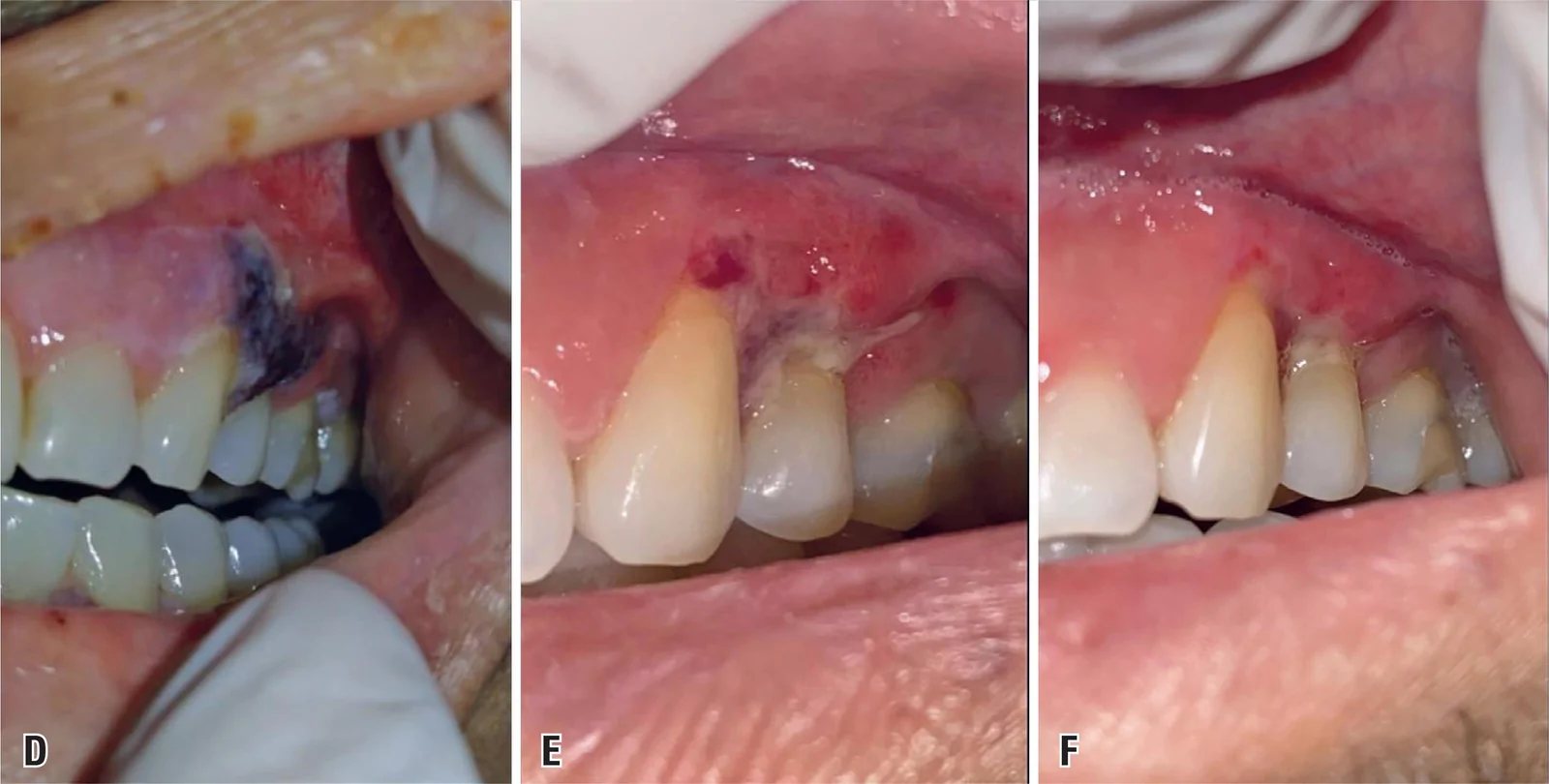
Clinical evolution of gingival lesions associated with *Pseudomonas aeruginosa* infection in a patient diagnosed with acute myeloid leukemia (AML), during hospitalization prior to hematopoietic stem cell transplantation. The diagnosis was confirmed by gingival scraping, microbial culture with antibiogram, and incisional biopsy of the upper left attached gingiva. The lower left canine gingiva was also affected. (D) Day 0: Initial presentation showing edema and a submucosal hematoma in the upper left gingiva. (E) Day 6: A necrotic appearance with tissue breakdown and fibrinopurulent exudate following biopsy. The patient was receiving systemic meropenem therapy plus alternate-day aPDT. (F) Day 8: Complete clinical resolution with preservation of gingival architecture and minimal irreversible tissue loss

All patients received systemic antibiotics selected according to antibiogram results in combination with alternating aPDT (methylene blue, 660nm, 4J/point) and PBM (660nm, 2 J/point). Lesions improved after the first few sessions, with complete resolution occurring within 3 to 16 days (median, 5 days). Pain, erythema, and exudate diminished early during treatment. Clinical assessment of pain, erythema, and exudate was performed through direct examination and serial photographic documentation without standardized clinical scales. Treatment was maintained until complete mucosal re-epithelialization was achieved, regardless of pain resolution. If the patient reported no pain but the lesion had not yet fully healed, sessions continued until complete tissue repair was confirmed. No adverse events or recurrence were observed. The combined protocol promoted rapid mucosal repair even in patients with severe thrombocytopenia and persistent leukopenia, suggesting that the therapeutic effect was primarily local and not dependent on systemic immune recovery.

## DISCUSSION

This case series demonstrates that the combination of aPDT and PBM, together with systemic antibiotic therapy, guided by microbiological culture and antibiogram, may represent an effective adjuvant strategy for oral *P. aeruginosa* infections in immunocompromised oncology patients. To date, this appears to be the first case series reporting the combined use of aPDT and PBM for managing oral *P. aeruginosa* lesions in cancer patients with microbiological confirmation. Available literature consists mostly of isolated case reports or studies involving infections in other anatomical sites, such as the skin, which are not directly applicable to the oral cavity.^(9,16,17)^ Although uncommon, these infections are clinically significant because *P. aeruginosa* exhibits broad antimicrobial resistance.^(1–4)^

All oral lesions achieved complete resolution. Although the observed clinical outcomes suggest favorable healing within 3 to 16 days, the absence of a control group precludes definitive conclusions regarding the isolated efficacy of the intervention. Median healing time was 5 days, considerably shorter than that reported in other clinical studies of *P. aeruginosa* infections, in which recovery often exceeded 15-20 days despite antibiotic therapy, as described by Lei et al. and Aspiroz et al.^(12,13)^ Furthermore, Eduardo et al. reported a case of oral *P. aeruginosa* infection refractory to systemic antibiotic therapy in which complete remission occurred only after 3 weeks of consecutive aPDT sessions.^(11)^ Compared with those reports, the healing times observed in this study suggest a potential benefit of the combined therapy. Individual clinical factors should also be considered: the patient with the longest healing time (16 days) had an extremely low leukocyte count (170/mm³), indicating severe immunosuppression that likely delayed tissue repair.

The rapid improvement and resolution of lesions in our patients support the synergistic mechanism of this combined therapy. This multimodal approach acts at different levels: systemic antibiotics provide systemic infection control, whereas topical aPDT generates reactive oxygen species following light activation of methylene blue, leading to bacterial cell wall destruction and reduction of local microbial burden. PBM, in turn, modulates inflammatory response, promotes angiogenesis, and accelerates epithelial repair.^(5–8,10)^ Alternating these modalities may enhance bacterial elimination while simultaneously promoting tissue healing, which is particularly beneficial in patients with hematologic malignancies or chemotherapy-induced cytopenias whose systemic healing capacity is often limited.

Culture results in this series revealed resistance patterns consistent with previous studies showing high resistance to fluoroquinolones and cephalosporins.^(4,5,7,9,11)^ However, they partially differ from Ahmed et al., who reported high resistance rates to aminoglycosides, particularly gentamicin (65%) and amikacin (32%) among *P. aeruginosa* isolates from oncology patients. Data regarding fluoroquinolones were more consistent: Ahmed et al. reported 48% resistance to ciprofloxacin compared with 33% in this study, reinforcing the limited therapeutic role of this drug class in severe OIs.^(4)^ Universal sensitivity to aminoglycosides reinforces the importance of culture-based antibiotic selection rather than empirical regimens.^(11,12,15)^ The predominance of negative blood cultures suggests that most infections were localized, emphasizing the advantage of local light-based therapy as an adjunct to systemic management.

Despite growing interest in combining aPDT and PBM for oral lesion management, few clinical studies have specifically addressed opportunistic bacterial infections such as those caused by *P. aeruginosa*. Most available publications focus on lesions of viral origin, graft-versus-host disease, or medication-related osteonecrosis of the jaw.^(5,6,9–11,16–18)^ Although in vitro studies have demonstrated the efficacy of aPDT against *P. aeruginosa* biofilms, clinical evidence remains limited.^(13)^ In this context, the present series contributes novel clinical evidence regarding the alternating application of aPDT and PBM in oncology patients with oral *P. aeruginosa* infection.

In this setting, the role of hospital-based dentists is critical for early diagnosis and monitoring of the clinical course of oral lesions associated with OIs. Microbiological swab sampling of oral lesions, although still underutilized in many services, enables precise pathogen identification and targeted antibiogram analysis, which is particularly important in immunosuppressed patients, in whom clinical presentation may be nonspecific and infection progression rapid and aggressive.^(3)^ The low positivity rate of blood cultures observed in this series, despite local culture confirmation of *P. aeruginosa*, highlights a key limitation of systemic diagnostic methods in localized OIs.

The therapy was well tolerated, with no adverse effects even in patients with severe thrombocytopenia and leukopenia, corroborating previous safety findings for PBM in oncologic settings.^(8,14,16)^ These results reinforce the potential of light-based therapy as a safe, rapid, and noninvasive adjunctive therapy for managing resistant oral infections, capable of promoting local healing even under unfavorable systemic conditions.

This case series provides relevant contributions to hospital-based clinical practice. One of its main strengths is the use of a clearly defined therapeutic protocol, supporting reproducibility in institutions with similar patient profiles. The study also reinforces the importance of microbiological culture and antibiogram testing for guiding more effective treatment. Nevertheless, some limitations must be considered. The small sample size limits generalizability and precludes robust statistical analyses. In addition, clinical heterogeneity among patients, including different cancer diagnoses and degrees of immunosuppression, may have influenced outcomes such as healing time.

Future prospective randomized controlled clinical trials are needed to validate the efficacy of combined aPDT and PBM as a therapeutic strategy for immunocompromised patients with opportunistic bacterial infections.

## CONCLUSION

Combined antimicrobial photodynamic therapy and photobiomodulation, together with systemic antibiotic therapy, guided by microbiological culture and antibiogram, resulted in rapid and complete healing of oral *P. aeruginosa* infections in immunocompromised oncology patients. This multimodal light-based approach proved safe, reproducible, and clinically valuable as an adjunct to systemic antibiotics, representing a promising therapeutic alternative for managing multidrug-resistant oral infections in complex oncologic settings. All cases achieved clinical resolution, with healing times comparable to or shorter than those previously reported in the literature. Local swab-based diagnosis and antibiogram testing enabled identification of high resistance rates to fluoroquinolones and cephalosporins, whereas aminoglycosides remained effective. Although the findings are promising, controlled studies are required to validate this approach for opportunistic bacterial infections.

## Data Availability

The datasets generated and analyzed during the current study are available from the corresponding author upon reasonable request.
